# Tailoring communication in consultations with women from high risk breast cancer families

**DOI:** 10.1038/sj.bjc.6600484

**Published:** 2002-08-27

**Authors:** E A Lobb, P N Butow, B Meiser, A Barratt, C Gaff, M A Young, J Kirk, G K Suthers, K Tucker

**Affiliations:** Medical Psychology Research Unit, Department of Psychological Medicine, University of Sydney, Sydney, New South Wales 2006, Australia; Department of Psychological Medicine, Royal North Shore Hospital, St Leonards, New South Wales 2065, Australia; Department Public Health and Community Medicine, University of Sydney, Sydney, New South Wales 2006, Australia; Victorian Clinical Genetics Services, Royal Children's Hospital, Parkville, Victoria 2337, Australia; Royal Melbourne Hospital, Parkville, Victoria 3050, Australia; Peter MacCallum Cancer Institute, Melbourne, Victoria 3000, Australia; Familial Cancer Service, Westmead Hospital, Westmead, New South Wales 2145, Australia; South Australian Clinical Genetics Service, Women's and Children's Hospital, North Adelaide SA 5000, Australia; Hereditary Cancer Clinic, Prince of Wales Hospital, Randwick, NSW 2031, Australia

**Keywords:** familial breast cancer, predictors, consultant communication

## Abstract

This multicentre study examined the influence of patient demographic, disease status and psychological variables on clinical geneticists/genetic counsellors (consultants) behaviours in initial consultations with women from high-risk breast cancer families. One hundred and fifty-eight women completed a pre-clinic self-report questionnaire. The consultations were audiotaped, transcribed verbatim and coded. Consultants did not vary their behaviour according to women's expectations. However, significantly more aspects of genetic testing were discussed with women who were affected with breast cancer (*P*<0.001), screening and management with unaffected women (*P*=0.01) and breast cancer prevention with younger women (*P*=0.01). Prophylactic mastectomy was discussed more frequently with women with medical and allied health training (*P*=0.02), and prophylactic oophorectomy with women affected with breast cancer (*P*=0.03), those in non-professional occupations (*P*=0.04) and with a family history of breast and ovarian cancer (*P*<0.001). Consultants used significantly more behaviours to facilitate understanding with women who were in non-professional occupations (*P*=0.04); facilitated active patient involvement more with women affected with breast cancer (*P*<0.001) and used more supportive and counselling behaviours with affected women (*P*=0.02). This study showed that patient demographics were more likely to predict consultants' communication behaviours than the woman's psychological status. Methods to facilitate assessment of psychological morbidity are needed to allow more tailored communication.

*British Journal of Cancer* (2002) **87**, 502–508. doi:10.1038/sj.bjc.6600484 www.bjcancer.com

© 2002 Cancer Research UK

## 

Previous research has documented consultants' reports of the cancer genetic services typically provided to women from high-risk breast cancer families. These include cancer risk assessment and education, facilitation of genetic testing, pre- and post-test counselling, provision of personally tailored cancer risk management recommendations, and psychosocial counselling and support services (Nh and Mrc, 1999).

A recent survey of Australian clinical geneticists and genetic counsellors identified the provision of individualised care as the single most important goal of genetic counselling (Lobb *et al*, 2001). These consultants emphasised the variability in women's current levels of expectations and needs when they attend genetic counselling and the importance of tailoring communication accordingly.

Studies that have examined levels of breast cancer genetics knowledge among women with a family history of breast cancer (Lerman *et al*, 1996, 1997; Hughes *et al*, 1997; Wonderlick and Fine, 1997; Cull *et al*, 1998; Bluman *et al*, 1999; Donovan and Tucker, 2000) have found wide variation in knowledge about many facets of genetic testing, including the cancer risks associated with different genes and different mutations and the effectiveness of interventions, for example screening, chemoprevention, or surgery for reducing risk (Geller *et al*, 1997; Audrain *et al*, 1998; Bluman *et al*, 1999).

Similarly, there is wide variation in the accuracy of women's perceptions of their likelihood of developing breast cancer. For example, the percentage of women who overestimated their risk after counselling has ranged from 14% to 89% (Evans *et al*, 1994; Lerman *et al*, 1995; Lloyd *et al*, 1996; Watson *et al*, 1999). Such over-estimation is associated with high levels of anxiety, and unless corrected, may lead to poorly informed decisions regarding breast cancer screening (Lerman *et al*, 1993; Alexander *et al*, 1996; Kash, 1996). Thus it is important to identify strategies which will optimise understanding and psychological adjustment, and these strategies are likely to vary according to the presenting characteristics of the women.

However, it is not known whether consultants tailor the consultation to the individual woman. No previous study has documented what actually happens in genetic counselling consultations with women from high-risk breast cancer families. This study assessed the process of genetic counselling for Australian women from familial breast cancer families and examined whether differing patient demographics, disease status (unaffected/affected) or psychological variables influence consultant communication.

## METHODS AND MATERIALS

### Participants

Women assessed to be at potentially high risk for the development of breast cancer because of their family history, and who were attending their first consultation in any one of 10 familial cancer clinics in four Australian States (New South Wales, Victoria, South Australia and Queensland), were included in the study. Women were quota-sampled according to whether or not they had previously had breast cancer. Women were considered ineligible for participation if they were unable to give informed consent, that is, if they were younger than 18 years or showed evidence of a severe mental illness. Individuals with limited literacy in English were also excluded because data collection was based on self-administered questionnaire.

A sample size of 158 women is sufficient to detect an association between patient characteristics and consultants' behaviours of a magnitude of 0.3, that is a small to medium effect size, at a 0.05 level of significance with a power of 80%.

### Procedure

This study is one component of a larger randomised controlled trial of providing women with an audiotape of their genetic counselling consultation, the results of which will be reported separately. Staff at each of the participating clinics invited women to participate in the study when they telephoned to make their appointment. If verbal agreement was obtained, women were mailed a self-administered questionnaire prior to their genetic consultation that they returned in a pre-paid envelope. The consultations were audiotaped and copies of the audiotape were retained for analysis. Ethics approval from 10 different ethics committees responsible for each of the participating clinics was sought and obtained prior to data collection.

### Measures

#### Demographic characteristics

Women were asked to provide details on age, education, occupation, marital status, language spoken at home, parents' country of birth, marital status, medical or allied health training, religious affiliation or spiritual belief and the number of biological children and sex and age of each child.

#### Cancer burden

Number of first- and second-degree relatives who had developed and/or died of breast or ovarian cancer were collected from study participants as a measure of ‘cancer burden’.

#### Breast cancer knowledge

An eight-item true–false measure assessed knowledge about breast cancer genetics. The scale is a revised version of a measure previously used in a study on the psychological impact of BRCA1 testing (Lerman *et al*, 1996). The scale has been found in previous studies to have moderate internal consistency with Cronbach's co-efficient alpha of 0.59 (Meiser *et al*, 2001).

#### Expectations

Women were asked to indicate on a five-point scale ranging from ‘not at all important’ to ‘very important’ their response to seven possible reasons for attending a genetic clinic and to similarly rate nine possible information topics they might want covered at their first appointment. This scale was developed for the purposes of this study and included items suggested by expert opinion, the literature and structured telephone interviews with at risk women. Whether expectations were met was determined by comparing for each woman what she expected and what she got according to the coded transcribed consultation.

#### Risk perception

Women were asked to indicate their perceived approximate lifetime risk (i.e. to age 80) of breast cancer (or, if affected, a second breast cancer) by choosing between seven response options ranging from 1–100%. A decision was made to code women's risk accuracy within categories, as risk estimates vary widely and often only a general risk category (e.g. high, medium or low) is given in the genetic counselling session. Participants' numerical estimate of life time risk was converted to a category according to the figures given in the Australian National Health and Medical Research Council Guidelines e.g. a potentially high risk category 25–80%; a medium risk category 12–25% and a low risk category 9–12% (Nh and Mrc, 1999).

#### Objective risk

This was determined by the figure given by the consultants in the consultation or the post consultation summary letter (all women received a figure in either of these communications). Participants' responses were deemed accurate if their risk estimate fitted within the risk category given by the consultant. If women were inaccurate it was determined whether they had under-estimated or over-estimated their risk of breast cancer.

#### Breast cancer-specific anxiety

This was measured using the Impact of Events Scale, a 15-item validated scale measuring intrusion and avoidance responses in relation to a specific stressor (Horowitz *et al*, 1979). In the current study the particular stressor was concern about being at risk of developing breast cancer for unaffected women and concern about developing a second cancer for affected women. In a previous validation study of women with a family history of breast cancer, the intrusion and the avoidance sub-scales have been found to be highly consistent with Cronbach's coefficient alpha of 0.84 and 0.91 respectively, and a test-retest reliability of *r*=0.80 (Thewes *et al*, 2001). Scores above 40 on either scale indicate a significant stress response.

#### General anxiety and depression

The 14-item Hospital Anxiety and Depression Scale has been found to be valid and reliable in detecting depression and anxiety in hospital medical outpatient clinics. It consists of two sub-scales of seven items assessing the level of anxiety and depression (Zigmond and Snaith, 1983). Scores range from 0–42. Questions have four response options, yielding scores ranging from 0–21 for each sub-scale. A score of higher than 10 on either sub-scale is an indication of clinical anxiety or depression, and scores from 8–10 on either sub-scale are indicative of ‘borderline’ anxiety and depression.

### Coding of transcripts of audio-tapes

A detailed coding system and coding manual for the transcribed audiotapes was devised. The coder's manual was developed to enable standardisation of the coding procedure and to facilitate calculation of inter-rater reliability.

The transcripts were coded to capture 10 aspects of the genetic counselling that encompassed (a) *Information giving behaviours*: (i) breast cancer genetics; (ii) genetic testing; (iii) family history and risk; (iv) prophylactic surgery; (v) breast cancer prevention; (vi) screening and management; and (b) *Process behaviours*: (vii) facilitating patient involvement; (viii) understanding; (ix) patient centredness and partnership building; and (x) supportive and counselling behaviours. These categories were based on (i) the National Health and Medical Research Council's Guidelines on the Familial Aspects of Cancer: A Guide to Clinical Practice (Nh and Mrc, 1999), (ii) an Australian survey of clinical geneticists/genetic counsellors describing their practice (Lobb *et al*, 2001) and (iii) studies that identified women's expectations of the genetic counselling session (Hopwood *et al*, 1993; Julian-Reynier *et al*, 1996, 1998; Hallowell *et al*, 1997; Michie *et al*, 1997; Veach *et al*, 1999; Brain *et al*, 2000).

Under each of these categories the content or behaviours that characterised that issue were identified. The presence or absence of each component was coded. Whether the woman or the genetic counsellor/geneticist initiated the content was noted and finally, the actual words used were recorded. An example of coding for an information giving behaviour is shown in Table 1 and a

**Table 1:**
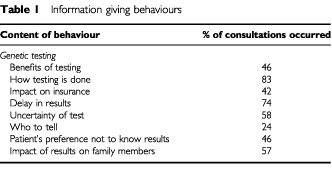
Information giving behaviours

process variable is shown in Table 2

**Table 2:**
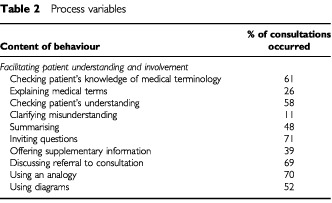
Process variables

. For example the category of *Genetic Testing* has eight component behaviours that were summed to provide a total score.

### Coding reliability

Three coders (including EL) were trained. Two coders re-coded a random 10% of their own consultation transcripts and 10% of the other coder's consultation transcripts to determine intra-and inter-rater reliability. There were 274 content coding variables for each consultation. The average inter-rater reliability over the 274 codes was 93% (range 67–100%) and the average intra-rater reliability was 92% (range 65–100%). The areas of highest agreement were information-giving categories: risk (96–100%); screening (89–99%); genetic testing (84–99%); prevention (91–100%); and there were lower levels of agreement on some consultant communication facilitation behaviours: facilitating communication (67–87%) and discussing psycho-social issues (69–76%). Thus interpretation of findings on these latter variables must be made with some caution.

### Statistical methods

Descriptive statistics (frequencies, means and medians) were used to summarise most of the data, including demographics and psychological status. Frequencies were calculated for each consultant behaviour. Total scores for the 10 pre-defined counselling categories were calculated by summing the component behaviours.

Univariate analyses exploring associations between the demographic, expectation, psychological, knowledge and family history predictors listed above and total scores for each counselling category were undertaken using parametric statistics (*t*-tests and Pearson's correlations) if the category total score was normally distributed and non-parametric statistics (Mann–Whitney U and Spearman's correlations) if the category total score was non-normally distributed. Some individual consultation behaviours are analysed by chi square analyses. Variables associated with the outcomes at *P*<0.25 (Hosmer and Lemeshoe, 1989) were included in multivariate analyses (linear regression if normally distributed, logistic regression with the dependent variable re-categorised above and below the median if non-normally distributed).

## RESULTS

Of the 231 women who met eligibility criteria, 11 women declined participation and 27 women did not attend their appointment. Of the remaining 193 women, 158 women completed baseline (and follow-up) questionnaires, for whom there was an audible audiotape of their consultation for verbatim transcription.

Of the final sample (*n*=158), 89 women were unaffected with breast cancer (56%) and 69 were affected (44%). The majority had a family history of breast cancer (77%) and almost a quarter had a family history of breast and ovarian cancer (23%). The median age of participants was 42 (range 19–79) s.d.=11.9. The majority of women were married or in a *de facto* relationship (76%). Over half the women were educated above year 12 (55%) and 57% were in professional or semi-professional occupations while 33% had some form of medical or allied health training.

Genetic counselling consultations were given by five clinical geneticists and two genetic counsellors. Three of the geneticists were male, two female, and all genetic counsellors were female. They were all very experienced in providing services to women from high risk families, and worked within a familial cancer clinic.

### Baseline characteristics

#### Women's knowledge of breast cancer genetics

Prior to the clinic visit, women gave a mean of five correct answers on the eight breast cancer genetics knowledge scale. Areas where fewer women gave correct answers concerned the role of male inheritance, the presence of more than one breast cancer pre-disposition gene and the effectiveness of bilateral mastectomy in reducing breast cancer risk. Multivariate analysis showed that women younger than 42 years (OR=0.469, 95%CI=0.247–0.889, *P*=0.02) and women in professional occupations (OR=0.490, 95%CI=0.262–0.917, *P*=0.03) gave more correct answers.

#### Women's risk perception at baseline

Just under half of unaffected women estimated their risk of breast cancer accurately. Of the women who were inaccurate, half underestimated and half overestimated their risk. Accuracy of risk perception prior to counselling was significantly associated with a higher educational level with 67% of women educated above year 12 being accurate compared to 33% of women educated below year 12 (χ^2^_1_=5.15, *P*=0.02).

#### Psychological Status

The majority of unaffected and affected women had normal scores on the Impact of Events Scale (intrusion and avoidance responses) prior to counselling. A small sub group of unaffected (10%) and affected (19%) women showed significant stress responses. Almost one-fifth of unaffected and affected women were clinically anxious and 18% of unaffected women and 28% of affected women were ‘borderline’ clinically anxious. Five per cent (5%) of both unaffected and affected women were clinically depressed and 8% of unaffected and 16% of affected women were ‘borderline’ clinically depressed. These results are not dissimilar to others reported for affected and unaffected women (Maguire *et al*, 1980; Meiser *et al*, 2000). Compared to the normal population, levels of psychological morbidity were not notably high for unaffected women, but anxiety was certainly higher in affected women and if probable cases are included, rates of clinically significant depression were also higher.

#### Women's expectations of the genetic counselling session

The vast majority of women came to genetic counselling to obtain information about prevention, surveillance and risk information for themselves and for their children. A large percentage (82%) expected a genetic test, while just under half the sample expected to discuss prophylactic surgery. About half the women expected to discuss their feelings and receive reassurance.

Table 3

**Table 3:**
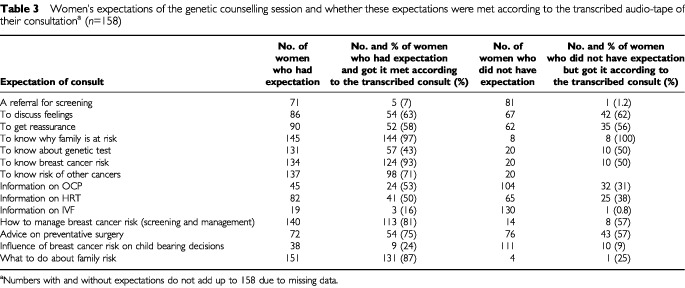
Women's expectations of the genetic counselling session and whether these expectations were met according to the transcribed audio-tape of their consultation^a^ (*n*=158)

represents a comparison between what women expected to receive from their genetic counselling session (as reported in their baseline questionnaire) and what they received (measured by examining the transcripts of their session). In two-thirds of consultations, the woman was asked her reason for attending genetic counselling (her agenda), offering the consultant an opportunity to assess expectations. Women's expectations to receive information about risk and aspects of preventative surgery were generally met. However, many women who did *not* expect this information also received it, as these topics appeared to be standard components of the genetic counselling consultation. Univariate statistical testing showed no relationship between baseline expectations concerning these topics and consultant behaviour. Similarly, there were no relationships between baseline expectations for discussion of feelings and reassurance, or for a genetic test, and consultant behaviours.

About half of the women who wanted information about hormone replacement therapy (HRT) and the oral contraceptive pill (OCP) received it, while about a third of the group who did not expect these topics to be covered nevertheless discussed them. Univariate tests revealed a significant relationship between expectations and consultation behaviour concerning OCP (χ^2^_1_=6.3, *P*<0.01). Very few women expected or received information about IVF. A larger number (38) expected to discuss the impact of breast cancer risk on child bearing decisions, and those with this expectation were more likely to discuss it (χ^2^_1_=5.1, *P*=0.05).

### Did consultants tailor their consultations according to demographic and psychological characteristics?

A summary of the significant findings of predictors of consultants' communication behaviours is shown in Table 4

**Table 4:**
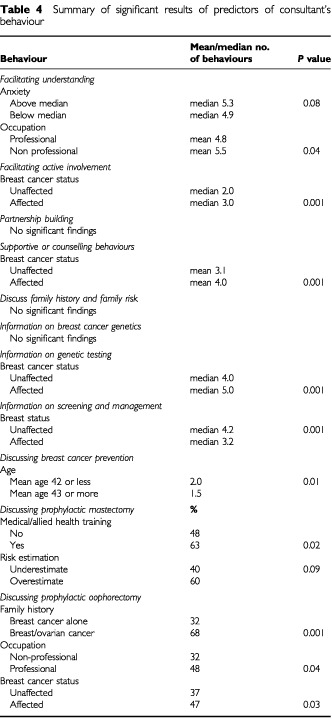
Summary of significant results of predictors of consultant's behaviour

. Multivariate analyses showed that consultants used significantly more behaviours that facilitated understanding with women who were in non-professional occupations (mean 5.5) than professional women (mean 4.8) (*t*=2.088, *P*=0.04), with a trend towards using more behaviours if the woman was anxious (median 5.3 compared to 4.9), (*t*=1.739, *P*=0.08). They used more behaviours to facilitate active patient involvement with women who were affected with breast cancer (median 3.0) compared to unaffected women (median 2.0), (OR=2.748, 95%CI=1.705–7.942, *P*=0.000).

Women's breast cancer status was found to be a significant predictor of supportive behaviours with consultants using more such behaviours with affected women (mean 4.0) than unaffected women (mean 3.1), (t_123_=−2.375, *P*=0.02).

No significant predictors were found of discussing family history and breast cancer genetics, which occurred in most consultations, nor of partnership building behaviours.

More aspects of genetic testing were discussed with affected women (median 5) than with unaffected women (median 4) (OR=3.593, 95%CI=1.786–7.225, *P*=0.000), while the reverse was true for information on screening and management (median of 4.2 issues discussed with unaffected women versus 3.2 with affected women) (*t*=−2.678, *P*=0.01). Non-medical strategies to prevent breast cancer (such as diet) were discussed more frequently with younger women aged 42 years or less, (median 2.0 behaviours) compared to older women (median 1.5) (*P*=0.01).

Prophylactic surgery was discussed in half of the consultations, but its discussion was initiated by the consultant in only a third of these consultations. Multivariate analyses showed that prophylactic mastectomy was more likely to be discussed with women who had some form of medical training (OR=6.872, 95%CI=1.290–36.597, *P*=0.02), and less with women who underestimated their breast cancer risk (OR=0.256, 95%CI=0.054–1.216, *P*=0.09). Prophylactic oophorectomy was discussed more with women who were affected with breast cancer (OR=2.126, 95%CI=1.054–4.289, *P*=0.03), worked in non-professional occupations (OR=0.470, 95%CI= 0.231–0.953, *P*=0.04), and who had a family history of breast and ovarian cancer (OR=4.568, 95%CI=2.017–10.345, *P*=0.000).

Levels of general anxiety and depression, cancer specific anxiety, knowledge and risk accuracy were not found to be associated with any consultant behaviour.

## DISCUSSION

This study is the first of its kind, and undertook a detailed analysis of transcripts of genetic counselling sessions with women from high-risk breast cancer families to identify the process and content of the consultation. In this paper, predictors of consultant behaviour were explored.

### Were women's expectations of genetic counselling met?

Previous authors have argued that the success of the genetic counselling session is dependent upon the counsellor accurately gauging the woman's needs and expectations. To avoid a mismatch in the consultant's and the woman's expectations, it has been recommended that the woman's agenda is discussed at the beginning of the counselling session (Michie *et al*, 1998). In this sample, the consultant elicited the woman's agenda in the majority of consultations (69%). However, a third of women were not given the opportunity to outline their informational and emotional needs. The impact of eliciting the woman's agenda and other consultant behaviours on patient outcomes is not the focus of this paper, but will be explored in another article.

The finding that the vast majority of women come to genetic counselling to obtain information about prevention, surveillance and risk information for themselves and for their children is similar to that reported by other studies (Julian-Reynier *et al*, 1996, 1998; Hallowell *et al*, 1997;). Four out of five women expected a genetic test, over a third expected a physical examination (33%) and just under half expected a referral for screening (43%).

Where the woman's agenda matched the consultant's (for information about breast cancer genetics and risk) most women received what they expected. However, women who were not expecting these facts were also likely to receive them, suggesting that consultants gave this information in a standard, rather than tailored manner. For example, half of women who had not expected to discuss their breast cancer risk did so.

Where expectations were not well matched (for example, for a genetic test, a screening referral and reassurance) the majority of women did not receive what they expected, and their expectations did not influence consultants' behaviour. However, women did appear more successful in communicating their expectations of fertility topics, with consultants appearing more responsive to their expectations. If women's expectations are unrealistic (for example, women can only receive a test if they have a living affected relative), it may be beneficial to discuss this when they make their genetic counselling appointment, to avoid unrealistic expectations. The impact of meeting women's expectations on their satisfaction with the encounter will be explored in another paper.

### Do consultants tailor the consultation according to demographic and psychological factors?

This study found that breast cancer status was a significant predictor of consultants' behaviours. For example, consultants used significantly more supportive and counselling behaviours with women who were affected with breast cancer and facilitated more active patient involvement with such women. Women with breast cancer are faced with an uncertain prognosis and many experience clinically significant levels of depression and anxiety (Fallowfield *et al*, 1990; Kissane *et al*, 1998). It is likely that consultants were responding appropriately to these high emotional distress levels in providing more support.

Affected women in this sample were significantly more likely to be psychologically distressed before the consultation than were unaffected women. However, levels of anxiety and depression prior to counselling were not independently associated with consultant behaviour, suggesting that consultants were depending more on their knowledge of breast cancer status, than on the individual's current state. The literature suggests that under-detection of patient distress is common in medical practice. For example Ford *et al* (1994) found that five oncologists' ability to accurately detect distress in 117 newly referred out-patients was low, with underestimation of distress most common. Specific strategies to explore emotionality may be required if consultants are to tailor their behaviour accordingly.

Doctor behaviours associated with greater patient disclosure of emotion have been identified in a number of studies. These include: open directive questions, focussing on and clarifying psychological issues, empathic statements, immediate response to patient cues, summarising, and making educated guesses about what the patient might be feeling (Goldberg *et al*, 1993; Maguire *et al*, 1996). Improving this aspect of consultants' behaviour may be important as it has been shown to have an impact on patient outcomes in medical consultations (Bertakis *et al*, 1991). Whether it is appropriate to raise complex emotional issues within the limited timeframe of a genetic consultation, and the level of intervention required, remain to be determined.

Similarly, consultants used significantly more behaviours that facilitated understanding (for example summarising, inviting questions, or using diagrams) with women who were in non-professional occupations, and there was a trend for consultants using more of these behaviours if the woman was anxious. This suggests they were responding appropriately to women whose understanding might be expected to be poorer. However, levels of knowledge and risk accuracy prior to counselling were not related to the level of detail provided about genetics, risk and screening, suggesting that these were not easy to assess within the consultation. As with emotional status, routine assessment of these features before the consultation, fed back to the consultant, might provide useful cues to the consultant on the level of detail required by each person. Several instruments to assess understanding are currently available (for example the measure used in the current study) that might prove useful for this purpose.

Consultants were also more likely to discuss genetic testing and prophylactic oophorectomy with affected women, and screening and management issues with unaffected women. Since mutation detection in Australia is initiated in affected women and unaffected relatives are only offered predictive testing once a family-specific mutation has been identified, it is not surprising that genetic testing is more frequently discussed with affected women. Nor is it surprising that prophylactic oophorectomy is more frequently discussed with affected women (even if their mutation status is unknown), since it is very likely that they are carriers of a mutation that may also increase their risk of ovarian cancer. By contrast, an unaffected woman with unknown mutation status has a less than one in two chance of being a carrier and thus her a priori risk of developing ovarian cancer is considerably lower. On the other hand, screening and preventive strategies, such as taking tamoxifen, are more relevant to unaffected women, and appropriately discussed more frequently with them.

The woman's occupation (and medical training) were also significant predictors of consultant behaviours, in particular, discussion of prophylactic surgery. Prophylactic surgery was most commonly raised by the woman herself, and it may be that those with medical training were more aware of mastectomy as an option, and therefore initiated the discussion.

Finally, consultants facilitated more active involvement in affected women. There may be a perception that affected women are more experienced in the medical encounter, and more familiar with decision making, and these women certainly have more decisions to make.

### Conclusions

This study has concluded that consultants tailor their information giving according to clinical factors, such as breast cancer status, but that many other features, such as women's expectations, their psychological state and knowledge, do not influence consultant behaviour. It is likely that these features are not easy to assess in the consultation, and strategies to more directly assess these features, such as through questionnaire or clinical questioning, will be required if consultants wish to tailor their consultation accordingly.
